# Fluorescence Polarization Immunoassay for the Determination of T-2 and HT-2 Toxins and Their Glucosides in Wheat

**DOI:** 10.3390/toxins11070380

**Published:** 2019-07-01

**Authors:** Vincenzo Lippolis, Anna C. R. Porricelli, Erminia Mancini, Biancamaria Ciasca, Veronica M. T. Lattanzio, Annalisa De Girolamo, Chris M. Maragos, Susan McCormick, Peiwu Li, Antonio F. Logrieco, Michelangelo Pascale

**Affiliations:** 1 Institute of Sciences of Food Production (ISPA), National Research Council of Italy (CNR), 70126 Bari, Italy; 2 Mycotoxin Prevention and Applied Microbiology Research Unit, Agricultural Research Service, U.S. Department of Agriculture, Peoria, IL 61604, USA; 3 Key Lab for Mycotoxins Detection, Oil Crops Research Institute of the Chinese Academy of Agricultural Sciences, Wuhan 430062, China

**Keywords:** fluorescence polarization immunoassay, T-2 toxin, HT-2 toxin, T-2 glucoside, HT-2 glucoside, wheat, validation study, screening method

## Abstract

T-2 and HT-2 toxins and their main modified forms (T-2 glucoside and HT-2 glucoside) may co-occur in cereals and cereal-based products. A fluorescence polarization immunoassay (FPIA) was developed for the simultaneous determination of T-2 toxin, HT-2 toxin and relevant glucosides, expressed as sum. The developed FPIA, using a HT-2-specific antibody, showed high sensitivity (IC_50_ = 2.0 ng/mL) and high cross-reactivity (100% for T-2 toxin and 80% for T-2 and HT-2 glucosides). The FPIA has been used to develop two rapid and easy-to-use methods using two different extraction protocols, based on the use of organic (methanol/water, 90:10, *v*/*v*) and non-organic (water) solvents, for the determination of these toxins in wheat. The two proposed methods showed analytical performances in terms of sensitivity (LOD 10 µg/kg) recovery (92–97%) and precision (relative standard deviations ≤13%), fulfilling the criteria for acceptability of an analytical method for the quantitative determination of T-2 and HT-2 toxins established by the European Union. Furthermore, the methods were then validated in accordance with the harmonized guidelines for the validation of screening methods included in the Regulation (EU) No. 519/2014. The satisfactory analytical performances, in terms of intermediate precision (≤25%), cut-off level (80 and 96 µg/kg for the two methods) and rate of false positives (<0.1%) confirmed the applicability of the proposed methods as screening method for assessing the content of these toxins in wheat at the EU indicative levels reported for T-2 and HT-2 toxins.

## 1. Introduction

T-2 toxin (T-2) and its deacetylated form HT-2 toxin (HT-2) are epoxy sesquiterpenoids, classified as type-A trichothecene mycotoxins, produced by several *Fusarium* species, mainly *F. langsethiae* and *F. sporotrichioides*, in cereal grains under cool and moist conditions in the field and after harvesting. Several studies report the incidence of T-2 and HT-2 mainly in oats, but also in other grains including barley, wheat, maize, rice and soybean, as well as in cereal-based products [1,2,3,4]. Being potent inhibitors of DNA, RNA and protein synthesis, T-2 and HT-2 can cause several adverse effects in both humans and animals [3,5]. Due to their toxicity and co-occurrence, the Panel on Contaminants in the Food Chain (CONTAM Panel) of the European Food Safety Authority (EFSA) established a group tolerable daily intake (TDI) of 100 ng/kg body weight per day for the sum of T-2 and HT-2 [3]. Although maximum permitted levels were not established for T-2 and HT-2, the European Union provided indicative levels for the sum of these toxins in cereals and derived products ranging from 15 to 2000 µg/kg [6] from which investigations should be performed to assess changes and trends in human and animal exposure. For unprocessed wheat samples, the indicative level for T-2 and HT-2 was 100 µg/kg (expressed as sum).

**Figure 1:**
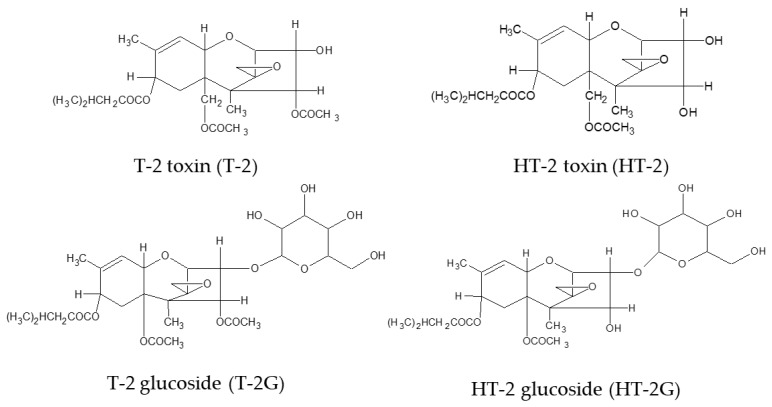
Chemical structures of T-2 and HT-2 toxins and their main modified forms T-2 and HT-2 glucosides.

Several modified forms of T-2 and HT-2 generated by fungi, plants and mammals were isolated and characterized, as reported in the EFSA Scientific Opinion [7]. Among the possible modifications for these toxins, conjugation with sugars, mainly with glucose, as glucopyranosides play an important role leading to the formation of T-2 glucoside (T-2G) and HT-2 glucoside (HT-2G). Figure 1 reports the chemical structures of T-2, HT-2, T2-G and HT-2G. The presence of T-2G and HT-2G in naturally contaminated cereals, including wheat, oat, maize and barley, have been reported by several authors [8,9,10,11] and recently reviewed [12]. Only limited toxicological data are available for T-2G and HT-2G, but they could also be toxic by releasing their aglycones, either during food processing or in the gastrointestinal tract after ingestion [7]. Although the CONTAM Panel found it appropriate to establish a group TDI for T-2 and HT-2 and their modified forms, they concluded that this assumption would determine an overestimation of risk for these toxins; therefore, based on new toxicity studies, the group TDI, only for T-2 and HT-2, was recently amended to 20 ng/kg body weight per day [7]. Moreover, an estimation of human and animal dietary exposure to T-2 and HT-2 has been recently carried out by EFSA. However, due to lack of data, the potential presence of the T-2/HT-2 modified forms was not considered, which could determine an underestimation of the real exposure [4]. For these reasons, the collection of analytical data on T-2 and HT-2, including modified forms, in food and feed commodities is highly needed using analytical methods with an appropriate sensitivity and accuracy [4,11]. Moreover, the availability of analytical methods able to detect simultaneously mycotoxins and their modified forms, even though expressed as the sum, is very useful especially in view of possible future requirements of European Regulations.

A large number of analytical methods are available for the determination of T-2 and HT-2 in cereals and derived products, based on high- or ultrahigh-performance liquid chromatography (HPLC and UHPLC) and gas chromatography (GC) [13,14,15]. Although these methods have high accuracy and sensitivity permitting the determination of T-2 and HT-2 at the indicative levels recommended by the European Commission [6], they cannot be used for the determination of relevant glycosylated forms. For this reason, LC-MS analysis is the most widely used approach for the simultaneous determination of T-2/HT-2 and relevant glucosides, which are also often detected together with other co-occurring mycotoxins, as well as their modified forms [11,16]. However, these analytical methods are expensive, time-consuming and require skilled personnel. For this reason, easy-to-use, rapid, cheap, high-throughput, robust and reliable analytical methods for the simultaneous monitoring of T-2 and HT-2 and relevant glucosides are in great demand for collecting more data and correctly evaluating the real exposure to these toxins. Moreover, in the development of rapid test kits for the detection of mycotoxins the use of non-organic solvents is especially encouraged to provide kits for users who are not familiar with the management and disposal of the organic solvents [17].

In the last decade, several rapid methods have been developed for the detection of T-2 and HT-2 in cereals and derived products, mainly immunochemical methods [13], such as enzyme-linked immunosorbent assays [18,19,20,21], lateral flow devices or dipsticks [22], fluorescence polarization immunoassays (FPIAs) [23,24], biosensors and immunochips [25,26] and, more recently, also methods based on the use of aptamers as alternative receptors [27,28]. None of these methods were developed for the simultaneous detection of T-2 and HT-2 and relevant glucosides. A monoclonal antibody was specifically designed and validated for T-2 and T-2G, and when used in ELISA, showed an IC_50_ in the low ng/mL range, suggesting its potential use for their simultaneous detection [29].

Among screening methods, FPIAs have been widely shown to be a useful tool for mycotoxin screening [30,31]. FPIAs are homogeneous immunoassays based on the competition between the analyte and tracer (fluorescent derivative of analyte) for a limited amount of antibody. The analyte content is determined by measuring the reduction of fluorescence polarization value, which is determined by the reduction of tracer molecules able to bind antibody in solution [30]. Several FPIA methods have been developed as screening tools for the determination of major mycotoxins, including aflatoxins, ochratoxin A, zearalenone, fumonisins, deoxynivalenol and T-2 and HT-2, in food matrices [30,31,32]. In particular, some FPIAs have been developed and validated for the determination of T-2 and HT-2, express as sum, in wheat, oats, barley and cereal-based products [23,24]. To date, no FPIAs and, more generally, no rapid methods are available for the simultaneous determination of T-2 and HT-2 and relevant glucosides.

For this reason, the aim of this study was to develop and validate an FPIA for the simultaneous determination, expressed as sum, of T-2, HT-2, T-2G and HT-2G in wheat. Two different extraction protocols, using organic (methanol/water) and non-organic (water) solvents, were optimized for the FPIA. The two developed methods based on the FPIA and using the two protocols have been validated in-house as quantitative methods, determining sensitivity, recovery and precision values. Furthermore, the two methods were validated through a single-laboratory validation protocol according to harmonized guidelines recently established by the Regulation (EU) No. 519/2014 [33]. The fitness-of-purpose of the FPIA was evaluated by calculating the method precision profiles and setting the screening target concentrations (STC) for false suspect rate and cut-off level to the EU’s indicative levels of the sum of T-2 and HT-2 in wheat [33].

## 2. Results and Discussion

### 2.1. Development of the FPIA

The antibody specific to the mycotoxin of interest and the tracer are key reagents in the development of a competitive FPIA for mycotoxin analysis. The most important features of the FPIA, such as incubation time, recovery, precision and sensitivity, are strictly related to the antibody/tracer combination used [30]. Binding experiments were performed, in buffer solution, by FP measurement to select the best antibody/tracer combination. Specifically, 48 different combinations were tested, derived from 12 monoclonal antibodies (i.e., ten specifics for T-2G, one for T-2 and one for HT-2) versus 4 different tracers (i.e., one T-2 and three HT-2 fluorescent derivatives). Table 1 reports the maximum values of polarization shift (ΔP_max_, maximum tracer-antibody binding) and optimized MAb concentrations obtained for each antibody/tracer combination. Among all MAb/tracer combinations tested, the highest bindings were observed for the four combinations obtained with anti-T2 and anti-HT2 versus T2-FL and HT2-FL_1a_, with ΔP_max_ ranging from 205 to 282 mP, and for fifteen combinations composed of anti-T2-glucoside antibodies and the four tracers with ΔP_max_ in the range of 133–280 mP (Table 1).

**Figure 2:**
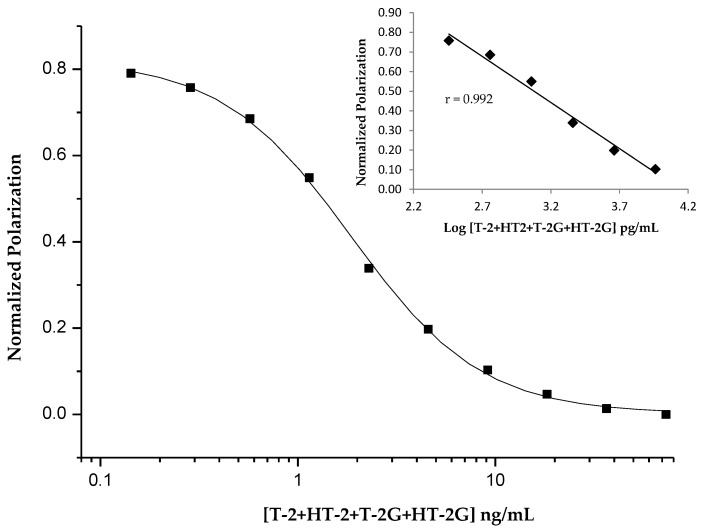
Normalized calibration curve of the selected FPIA obtained with mixed standard solutions of T-2, HT-2, T2-glucoside and HT2-glucoside (expressed as sum, ratio 1:1:0.5:0.5) in PBS-A solution ([Anti-HT2] = 8 µg/mL; [HT2-FL_1a_] obtained after dilution 1:3000 (*v/v*) of the stock solution, see Table 1). The FP linearity range vs. log [T-2 + HT-2 + T-2G + HT-2G] is reported in the insert. Values of the *x*-axes are the toxin concentrations in the final test solution.

All these selected combinations in the optimized concentrations were tested for competitive FPIAs with mixed T-2, HT-2, T-2G and HT-2G (ratio 1:1:0.5:0.5) standard solutions in different ranges of concentrations. Although all T2-glucoside MAbs exhibited suitable cross-reactivity for T-2, HT-2 and HT-2G, the assays showed poor sensitivity, with IC_50_ ≥ 12.2 ng/mL. The FPIA performed by Anti-T2/T2-FL and Anti-T2/HT2-FL_1a_ showed an acceptable sensitivity, with IC_50_ ≥ 4.0 ng/mL, but a low cross-reactivity for HT-2, T-2G and HT-2G ranging from 41 to 63%. On the other hand, the FPIA performed by using the Anti-HT2/HT2-FL_1a_ combination showed good sensitivity, with IC_50_ = 2.0 ng/mL and a high cross-reactivity of 100% for T-2 and 80% for T-2G and HT-2G (Figures S1–S4 report the calibration curves for the single toxins). Figure 2 reports the calibration curve for mixed standard solutions of T-2, HT-2 and their glucoside forms in the concentration range of 0.1–73.2 ng/mL (expressed as the sum of the toxins). An incubation time of 5 min was selected as optimal for this competitive immunoassay. The combination of Anti-HT2/HT-2FL_1a_ was then selected as the antibody/tracer combination to use for further development and validation of the assay.

### 2.2. Testing of Extraction Protocols and Evaluation of Matrix Effects

Two rapid extraction protocols based on the use of organic (methanol/water, 90:10, *v*/*v*, Protocol A) and non-organic (water, Protocol B) solvents were tested for the FPIA. Preliminary experiments, based on LC-MS analysis of raw extracts, were performed to assess the reliability in terms of accuracy of the protocols for the simultaneous extraction of T-2, HT-2, T-2G and HT-2G from spiked wheat samples at two levels: 300 and 600 μg/kg (expressed as sum). In the case of Protocol A, mean recoveries ranged from 110 to 119% for single toxins and from 112 to 115% for total toxins (expressed as sum). Relative standard deviations were lower than 7% for all tested toxins. In the case Protocol B, mean recoveries ranged from 88 to 106% for T-2G and HT-2G and from 163 to 192% for HT-2, with RSD lower than 18%. T-2 was not detected in any of the tested replicates at either spiking level. The absence of T2, together with the high recoveries for HT-2, was attributed to de-acetylation processes as a result of cereal carboxylesterases inducing the complete conversion of T-2 into HT-2 in water-based extracts, as previously observed [34]. Moreover, recoveries obtained for total toxins, expressed as sum, were between 88% and 98%, with RSD lower than 9% for both protocols. These results show the potential applicability of the tested extraction protocols for the FPIA, aiming to determine the sum of these toxins.

Studies to evaluate the presence of matrix interferences by FPIA using different amounts of matrix equivalent (5, 10 and 20 mg) indicated that no significant differences were observed between slopes (t_calc_ < 2.306; *p* < 0.05) and positions (t_calc_ < 2.262; *p* < 0.05) of the regression lines obtained with mixed standard solutions and those obtained with spiked diluted extracts for both optimized protocols (Protocol A and Protocol B, calibration curves were reported in Figure S5). These results indicated the total absence, up to the tested amounts, of detectable matrix effects for the developed FPIAs that could produce an overestimation of toxins content.

### 2.3. Validation of the Methods

To obtain a comprehensive analytical performance profile, the two methods based on the developed FPIA and using the two extraction protocols were validated in-house, both as quantitative methods and as screening methods.

Limit of detection (LOD) and limit of quantification (LOQ) of 10 and 15 µg/kg, respectively, were obtained for the FPIA using both extraction protocols (Protocol A and B) and analyzing 20 mg of matrix equivalent. These results indicated that the sensitivity of the methods was suitable for the quantitative determination of the target toxins at levels far below the indicative level suggested by the European Commission for the sum of T-2 and HT-2 in unprocessed wheat (i.e., 100 µg/kg). Recoveries (%) and repeatability (relative standard deviation, RSD, %) for the FPIA using both extraction protocols from wheat samples spiked with T-2, HT-2, T-2G and HT-2G in the range 50–200 µg/kg (expressed as sum) are reported in Table 2.

Mean recoveries for the FPIA using Protocol A ranged from 92 to 102%, with RSDs lower than 13%, whereas mean recoveries for FPIA using Protocol B were in the range 89–98%, with RSDs lower than 7%. Overall mean recoveries were 97 and 92% for FPIA using Protocols A and B, respectively. The values of recoveries and precision obtained for the developed FPIAs fulfil the criteria of acceptability for an analytical method for the quantitative determination of the native forms fixed by the European Commission [33].

Single-laboratory validation of the two developed methods was performed in-house over 5 different days in accordance with the harmonized guidelines for screening methods established by the Regulation (EU) No. 519/2014 by determining the precision profile of the method, the cut-off level and the false suspect rate. The summary results of the statistical assessment for blank samples and samples artificially contaminated at screening target concentration (STC, 100 µg/kg as the sum of T-2, HT-2, T-2G and HT-2G) are presented in Table 3. The STC value was set at the EU indicative levels of the sum of T-2 and HT-2 in wheat. The mean values of the test responses for the sum of these toxins were 115 and 104 µg/kg for samples at STC and 12 and 21 µg/kg for blank samples, for FPIA with Protocol A and Protocol B, respectively. Depending on the method used, relative standard deviation of the repeatability (RSD_r_) and relative standard deviation of intermediate precision (RSD_RI_) ranged from 5 to 13% for STC samples and from 14 to 25% for blank samples. The calculated cut-off levels were 96 and 80 µg/kg, for FPIA with Protocol A and Protocol B, respectively, and the rate of suspect results for blank samples was in both cases less than 0.1%.

**Figure 3:**
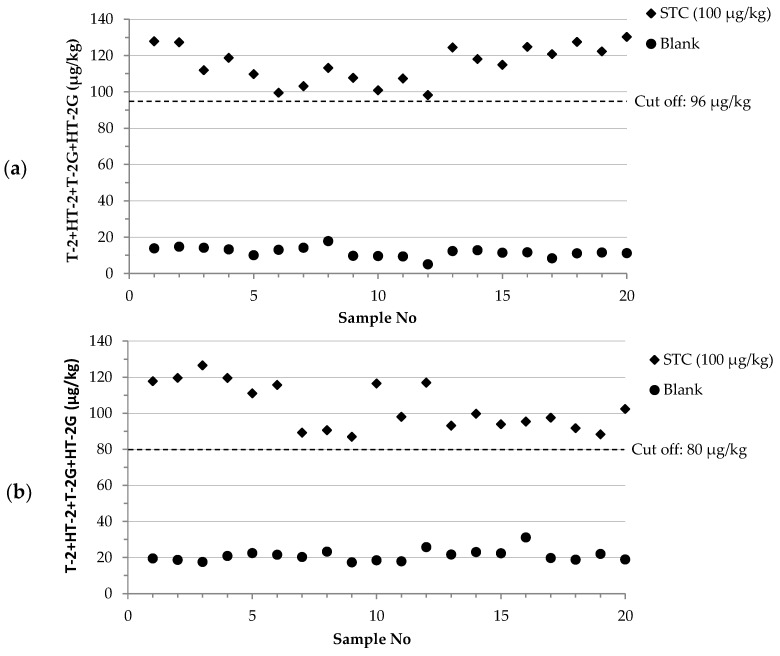
Contents of T-2, HT-2, T-2G and HT-2G (expressed as sum) of 20 artificially contaminated wheat samples at STC (100 µg/kg) and of 20 blank wheat samples analyzed under repeatability conditions on 5 different days: (**a**) FPIA using protocol A; (**b**) FPIA using protocol B.

Moreover, Figure 3 shows the graphical representation of the results, reporting the toxin contents obtained for the 20 artificially contaminated samples at STC and the 20 blanks, analyzed over 5 different days together with the calculated cut-off levels for the FPIA using Protocol A (Figure 3a) and Protocol B (Figure 3b). A complete separation between blanks and spiked samples at STC was observed for both methods, demonstrating their ability to discriminate between these two groups of samples. This aspect was also confirmed by the low rate of suspect results (less than 0.1%). The overall results confirmed the applicability of both methods for the simultaneous determination of T-2, HT-2, T-2G and HT-2G (expressed as sum) in wheat samples at the indicative level reported at EU level for the native forms T-2 and HT-2 from uncontaminated samples.

Moreover, there are several advantages to using a solvent-free extraction (Protocol B). Removal of organic solvents from the extraction is safer, because it reduces exposure of the analyst. It is also less expensive, because the cost associated with management of solvent waste (such as storage and disposal) are eliminated. Finally, eliminating solvents facilitates use in a wider variety of settings outside of the traditional laboratory.

## 3. Conclusions

A rapid FPIA for the simultaneous determination of T-2, HT-2, T-2G and HT-2G, expressed as their sum, was developed for the first time, by testing 12 monoclonal antibodies and 4 tracers. Two alternative extraction protocols, using organic and non-organic solvents, were optimised for the quantitative determination of these toxins in wheat. The main advantages of the developed methods were ease of use and speed (total time less than 15 min). The main disadvantage was the inability to determine the content of the individual toxins. The methods when validated as quantitative methods showed analytical performances, in terms of recovery (92–97%) and precision (RSDs ≤ 13%) that fulfilled the EU criteria for acceptability of an analytical method for the determination of native forms. Furthermore, the developed methods were also validated according to EU harmonized guidelines for the single-laboratory validation of screening methods. The satisfactory analytical performances, in terms of precision under repeatability (5–16%), intermediate precision (10–25%), cut-off level (80 and 96 µg/kg for the two methods) and rate of false positives (<0.1%), confirmed the applicability of the proposed methods based on the FPIA as screening methods for assessing the content of T2, HT2, T2G and HT2G in wheat at EU indicative levels reported for the native forms. Additional advantages of the FPIA methods were low-cost, portability, amenability to automation, and the use of environmentally friendly extraction procedures. These advantages make the developed FPIA methods useful and robust tools for high-throughput screening of these toxins in wheat, without the need for well equipped laboratories or personnel with a high level of technical skill.

## 4. Materials and Methods

### 4.1. Chemicals and Reagents

Acetonitrile and methanol were reagent grade or better and were purchased from Carlo Erba Reagents (Milan, Italy). Ultrapure water was produced by a Milli-Q^®^ Direct system (Merck KGaA, Darmstadt, Germany). T-2 and HT-2, sodium chloride (NaCl), sodium azide (NaN_3_), phosphate buffer saline (PBS) and ovalbumin (OVA) were purchased from Sigma-Aldrich (Milan, Italy). Ten monoclonal antibodies (MAbs) specific for T-2G (clones 1–2, 1–3, 1–4, 2–5, 2–11, 2–13, 2–16, 2–17, 2–21 and 2–44) were produced by US Department of Agriculture-Agricultural Research Center (USDA-ARS, Peoria, IL, USA) and have been described in Maragos et al. [29]. A Mab specific for T-2 (clone 1) was produced by Chinese Academy of Agricultural Sciences—Oil Crops Research Institute (Wuhan, China) and has been described by Zhang et al. [35]. A Mab specific for HT-2 (clone H10-A10) was purchased from University of Natural Resources and Life Science of Vienna—Department for Agrobiotechnology IFA-Tulln (Tulln, Austria). T-2 glucoside and HT-2 glucoside, both as α-anomers, were produced by USDA-ARS and have been described by McCormick et al. [36]. Glass culture tubes (10 × 75 mm) were obtained from VWR International (Milan, Italy). Paper filters (No. 4) and glass microfiber filters (GF/A) were purchased from Whatman (Maidstone, UK).

### 4.2. Preparation of Immunoassay Reagent Solutions

T-2 and HT-2 stock solutions, at the concentration of 1 mg/mL, were prepared by dissolving solid commercial toxins in acetonitrile. T-2G and HT-2G stock solutions were prepared at the concentration of 1 mg/mL in acetonitrile. Diluted T-2, HT-2, T-2G and HT-2G solutions were prepared in acetonitrile at the concentration of 20 µg/mL. Mixed standard solutions of T-2, HT-2, T-2G and HT-2G (ratio 1:1:0.5:0.5) were prepared in acetonitrile and PBS-A (PBS, 10 mM, pH = 7.4, containing 0.1% of NaN_3_) at the concentration of 10 and 3 µg/mL, respectively. The mixed standard solution in acetonitrile was used for spiking experiments in the LC-MS analysis to evaluate the extraction efficiency of the two alternative protocols. The mixed standard solution in PBS-A was used to prepare FPIA calibration curves. T-2 and HT-2 tracers (T2-FL: fluorescein-labelled T2 toxin; HT2-FL_1a_ and HT2-FL_1b_: monosubstituted fluorescein-labelled HT2 toxin, arbitrarily ascribed to each isomeric product; HT2-FL_2_: bi-substituted fluorescein-labelled HT2 toxin tracers) were prepared according to the procedure reported by Lippolis et al. 2011 [23], who also reported their chemical structures. Tracer working solutions were prepared daily by diluting the relevant stock solutions in methanol at the concentration providing a total fluorescence intensity equal to 3-fold the blank signal measured for the assay buffer (PBS-A). In the case of the selected tracer (HT2-FL_1a_) for the developed FPIA the dilution ratio was 1:3000 (*v*/*v*).The twelve monoclonal antibodies were diluted with PBS-OVA (PBS-A, containing 0.1% of OVA) according to the experiments reported in the Section 4.4 (FPIA analysis).

### 4.3. Sample Preparations

Durum wheat samples of different cultivars were collected from several fields in Italy. Samples were milled by the Ultra Centrifugal Mill ZM 200 (Retsch Technology GmbH, Hann, Germany) equipped with a 500-µm sieve. Two different extraction protocols, using organic (methanol/water) and non-organic (water) solvents, were tested. In particular, in the first approach (Protocol A), an aliquot (50 g) of wheat, added together with NaCl (1 g), was extracted with 100 mL of methanol/water (90/10, *v*/*v*) by blending at high speed for 3 min using a Steril Mixer 12 blender (VWR International). After filtering extracts through the filter paper, they were diluted with a 4% NaCl solution (ratio 1:5, *v*/*v*) and left to rest for 5 min. The dilution with 4% NaCl solution was carried out in order to reduce the percentage of methanol in solution and to precipitate interfering compounds, which may contribute to increasing the matrix effect. The diluted extracts were filtered by glass microfiber filters and analyzed by FPIA. In the case of the second approach (Protocol B), an aliquot of ground wheat samples (10 g) was extracted with 100 mL of water by blending at high speed for 3 min by Steril Mixer 12 blender. To evaluate the performances of both protocols for the simultaneous extraction of the tested toxins, recovery experiments were performed in triplicate by spiking uncontaminated (blank) wheat samples with a mixed T-2, HT-2, T-2G and HT-2G (ratio 1:1:0.5:0.5) spiking solution in acetonitrile at levels of 300 and 600 μg/kg. Extracts of both protocols (A and B) were filtered through a filter paper and a glass microfiber filter and subsequently analyzed by LC-MS analysis according to the experimental conditions reported in Lattanzio et al. 2012 [8].

### 4.4. FPIA Analysis

All FP measurements were carried out in the glass culture tubes and using a portable reader (Sentry^®^ 100, Diachemix Corporation, Milwaukee, WI, USA) with excitation and emission wavelengths of 485 and 535, respectively. Preliminary FP measurements were performed in buffer solution in order to find the best combination antibody/tracer to be used in the FPIA. In particular, the binding ability of the 12 MAbs was tested versus the 4 synthesized tracers by measuring the polarization shift (∆P = mP_MAb_ − mP_tracer_) observed between the test PBS-A solution containing the tracer (at the optimized concentration, see Table 1) and the test solution after adding the MAb working solutions at different concentrations and incubating in the range 0–10 min. In particular, the MAb working solution were 47.5–190 µg/mL for clone 1–2, 3.95–79 µg/mL for clone 1–3, 5.2–104 µg/mL for clone 1–4, 17.8–89 µg/mL for clone 2–5, 29.5–118 µg/mL for clone 2–11, 33.5–134 µg/mL for clone 2–13, 18.0–90 µg/mL for clone 2–16, 30.0–120 µg/mL for clone 2–17, 33.0–132 µg/mL for clone 2–21, 15.5–155 µg/mL for clone 2–44, 0.20–200 µg/mL for anti-T2 clone 1, 1.48–39.4 µg/mL for anti-HT2 clone H10-A10. For each Mab/tracer combination, the optimized MAb concentration corresponded to the lowest concentration providing the maximum value of ∆P (∆P_max_).Competitive FPIAs were carried out with mixed (ratio 1:1:0.5:0.5) standard solutions, in different concentration ranges, by using the nineteen selected antibody/tracer combinations (indicated in Table 1, using an arbitrary cut-off for the ∆P_max_ of 130 mP). In particular, the assays were carried out by adding 850 µL of PBS-A, 100 µL of antibody working solution and 50 µL of mixed T-2, HT-2, T-2G and HT-2G standard solution in a test tube. The polarization value of this test solution, previously mixed by vortex, was measured and used as the blank. An aliquot (25 µL) of tracer working solution (at the optimized concentration, see Table 1) was added and the solution was gently mixed by vortex. After incubating (in the range 0–10 min) the polarization value, expressed in millipolarization units (mP), of the solution was measured. Competitive FPIAs were also performed using the selected antibody/tracer combination (clone H10-A10/HT2-FL_1a_) and standard solutions of the single toxins T-2, HT-2, T-2G and HT-2G to determine their midpoint concentrations (IC_50_).The cross-reactivity of the selected monoclonal antibody (clone H10-A10) has already been tested for structurally related toxins (deoxynivalenol, 3-acetyl-DON, 15-acetyl-DON, diacetoxyscirpenol, neosolaniol and nivalenol) and mycotoxins frequently occurring in wheat (ochratoxin A and zearalenone), showing a very low cross-reactivity for neosolaniol (CR% = 0.12%) and no cross-reactivity for all other tested toxins [23].

The developed FPIA were carried out by adding and mixing 700 µL of PBS-A, 100 µL of antibody working solution (clone H10-A10, 8 µg/mL), 200 µL of filtered extract for both protocols (Protocol A and Protocol B, equivalent to 20 mg of matrix) or 50 µL of mixed T-2, HT-2, T-2G and HT-2G standard solution. After reading the blank using an FP reader, 25 µL of tracer working solution (HT2-FL_1a_, dilution 1:3000, *v*/*v* of the stock solution) was added, and the final solution was mixed and incubated for 5 min. The polarization value after incubation was measured. The measured polarization values were normalized to fit in the range 0–1, the equation Y_obs_ = (mP_obs_ − mP_0_)/(mP_1_ − mP_0_) was used, where mP_obs_, mP_0_ and mP_1_ are the polarization of the test solution, of an antibody-free control solution and of a toxin-free control solution, respectively, and Y_obs_ is the normalized result for the test solution [30]. The content of T-2, HT-2, T-2G and HT-2G, expressed as sum, in the wheat extracts was determined by using the measured normalized polarization values and the FPIA calibration curves in the toxins concentration range 5.85–187.5 ng/mL.

### 4.5. Evaluation of Matrix Effects

The presence of matrix effect on the developed FPIA was evaluated for both protocols (Protocol A and Protocol B). In particular, diluted extracts of blank wheat were spiked at different T-2, HT-2, T-2G and HT-2G levels in the concentration range 5.85–187.5 ng/mL (expressed as sum) at different amount of matrix equivalent analyzed, i.e., 5, 10 and 20 mg. Calibration curves determined by using either standard solutions or spiked diluted extract of uncontaminated wheat samples were compared.

### 4.6. Validation as Quantitative Methods

The developed methods were validated in-house as quantitative methods, with their performances being evaluated in terms of sensitivity, recovery and repeatability. Experiments were carried out to determine the sensitivity of the FPIA using both protocols (A and B). In particular, limits of detection (LODs) of the FPIAs were calculated from the mean FP signals of representative uncontaminated wheat samples (*n* = 10, wheat samples of different cultivars) minus 3 standard deviations of the mean signal. Limits of quantification (LOQs) were calculated by determining the lowest amount of measured toxins (expressed as sum) that was quantitatively determined by the calibration curve within the linearity range of the FPIA.

Recovery experiments were performed by spiking, in triplicate, blank wheat samples at levels of 50, 100 and 200 µg/kg and subsequently analyzing them by FPIA using both optimized extraction protocols (Protocol A and Protocol B). This contamination range was selected in order to include the indicative level provided by the European Union for T-2 and HT-2 (100 µg/kg). Samples were left overnight at room temperature to allow solvent evaporation prior to the FPIA analysis.

### 4.7. Validation as Screening Methods

The developed methods were also validated through single-laboratory validation according to harmonized guidelines for screening methods established by the European Commission in the Regulation (EU) No. 519/2014 [33]. The fitness-of-purpose of the FPIAs was evaluated by calculating the method precision profiles and setting the screening target concentrations [33] for the false suspect rate and the cut-off level to the EU’s regulatory/indicative levels of the relevant native forms in durum wheat. The validation design required 2 sample sets: (1) 20 positive control samples, namely, wheat samples fortified with the mycotoxins at the STC (100 µg/kg for the sum of T-2, HT-2, T-2G and HT-2G), and (2) 20 negative samples (blank wheat samples). The measurements for each validation level (STC and blank) were evenly distributed over 5 different days, resulting in 4 independent analyses per day. Cut-off levels were calculated from the results obtained for the 20 samples spiked at the STC by using the equation provided in the Regulation (EU) No. 519/2014:

Cut-off = R_STC_ − *t*-value_(0.05)_ × SD_STC_ (1)

where R_STC_ is the mean response of the spiked samples at STC; *t*-value_(0.05)_ is the one tailed t-value for a rate of false negative results of 5% (i.e., 1.729 for 19 degrees of freedom and 20 replicates); the SD_STC_ is the standard deviation of intermediate precision (which is the sum of repeatability and between-day variability) at STC calculated by one-way ANOVA (*p*-value = 0.05). The false suspect rate is determined on the base of the results obtained for 20 blanks and the calculated cut-off by calculating the t-value from the following equation:

*t*-value = (mean_blank_ − cut-off)/SD_blank_ (2)

where mean_blank_ is the mean response of the 20 blank samples; SD_blank_ is the standard deviation of the intermediate precision of blank samples. From the calculated t-value, the rate of false suspect results for a one-tailed distribution was calculated as indicated in Section 4.8.

### 4.8. Statistical Analysis

FPIA data were fit to linear or sigmoidal equations with Origin software version 6.0 (OriginLab Corporation, Northampton, MA, USA, 1999) using the unweighted least-square method. For the sigmoidal fit, the equation used was of the form y = A_2_ + [A_1_ − A_2_/1 + (x/x_0_)^P^]. Here, A_1_ and A_2_ represent the initial value (left horizontal asymptote) and the final value (right horizontal asymptote), respectively, while x_0_ represents the inflection point (center), and P represents the power. In the experiments to measure matrix effects, the linear regression curves were compared using parallelism and position statistical tests [37]. In the recovery experiments, which used three spiking levels, the homogeneities of the variances and the homogeneities of the means were compared using Barlett’s test and one-way ANOVA (*p*-value = 0.05), respectively. Results from experiments from the single-laboratory validation studies were subjected to one-way ANOVA (*p*-value = 0.05) using the Microsoft Excel add-on. The rate of false suspect results relative to the calculated t-value from a one-tailed Student’s T Distribution was obtained using the spreadsheet function “TDIST”.

## Figures and Tables

**Table 1:** Maximum value of polarization shift (∆P_max_) obtained at the optimized antibody concentrations for each antibody/tracer combination.

MAb	Clone	[MAb] (µg/mL)	∆P_max_ (mP) ^1^			
			T2-FL (Dilution 1:3600) ^2^	HT2-FL_1a_ (Dilution 1:3000) ^2^	HT2-FL_1b_ (Dilution 1:400) ^2^	HT2-FL_2_ (Dilution 1:3600) ^2^
Anti-T2G	1–2	190	138 ^3^	-	59	57
	1–3	40	280 ^3^	39	262 ^3^	159 ^3^
	1–4	104	225 ^3^	28	253 ^3^	176 ^3^
	2–5	89	116	53	246 ^2^	27
	2–11	118	144 ^3^	18	50	18
	2–13	134	191 ^3^	23	28	19
	2–16	90	200 ^3^	37	82	21
	2–17	120	129	27	156 ^3^	18
	2–21	132	159 ^3^	22	14	16
	2–44	155	176 ^3^	133 ^3^	246 ^3^	49
Anti-T2	1	6	282 ^3^	217 ^3^	93	111
Anti-HT2	H10-A10	8	205 ^3^	230 ^3^	122	20

^1^ ∆P_max_ = mP_MAb_ − mP_tracer_; −∆P_max_ < 10mP; ^2^ Optimised dilution (*v*/*v*) of the stock solutions providing a total fluorescence intensity equal to 3-fold the blank signal measured for PBS-A; ^3^ selected antibody/tracer combinations.

**Table 2:** Average recoveries for T-2, HT-2, T-2G and HT-2G (expressed as sum) and relative standard deviations from spiked wheat obtained by FPIA using protocol A and B.

Spiking Levels (µg/kg)	FPIA			
	Protocol A		Protocol B	
	Recovery	RSD ^1^ (%)	Recovery	RSD ^1^ (%)
50	102	13	89	7
100	92	5	98	6
200	96	4	89	6
Overall average	97	9	92	7

^1^ RSD, relative standard deviation (*n* = 3 replicates).

**Table 3:** Statistical performances of the single-laboratory validation over 5 days of the FPIA for the determination of T-2, HT-2, T-2G and HT-2G (expressed as sum) with blank and artificially contaminated (at the screening target concentration of 100 µg/kg) wheat samples. Cut-off levels and rate of false suspect results were calculated according to the Regulation (EU) No. 519/2014.

Performances	Protocol A		Protocol B	
	Blank	STC ^1^ (100 µg/kg)	Blank	STC ^1^ (100 µg/kg)
Mean value ^2^ (µg/kg)	12	115	21	104
RSD_r_^3^ (%)	16	5	14	9
RSD_RI_^4^ (%)	25	10	16	13
Cut-off level		96		80
Rate of false suspect results (%)	<0.1		<0.1	

^1^ STC, screening target concentration; ^2^ The mean value of the total content of T-2, HT-2, T-2G and HT-2G (µg/kg, expressed as sum) (*n* = 20 replicates); ^3^ RSD_r_, relative standard deviation of the repeatability; ^4^ RSD_RI_, relative standard deviation (intermediate precision).

## Supplementary Materials

The following are available online at https://www.mdpi.com/2072-6651/11/7/380/s1, Figure S1: Normalized calibration curve of the selected FPIA obtained with mixed standard solutions of T-2 toxin (T-2) in PBS-A solution ([Anti-HT2] = 8 µg/mL; [HT2-FL_1a_] obtained after dilution 1:3000 (*v*/*v*) of the stock solution). Figure S2: Normalized calibration curve of the selected FPIA obtained with mixed standard solutions of HT-2 toxin (HT-2) in PBS-A solution ([Anti-HT2] = 8 µg/mL; [HT2-FL_1a_] obtained after dilution 1:3000 (*v*/*v*) of the stock solution). Figure S3: Normalized calibration curve of the selected FPIA obtained with mixed standard solutions of T-2 glucoside (T-2G) in PBS-A solution ([Anti-HT2] = 8 µg/mL; [HT2-FL_1a_] obtained after dilution 1:3000 (*v*/*v*) of the stock solution). Figure S4: Normalized calibration curve of the selected FPIA obtained with mixed standard solutions of HT-2 glucoside (HT-2G) toxin in PBS-A solution ([Anti-HT2] = 8 µg/mL; [HT2-FL_1a_] obtained after dilution 1:3000 (*v*/*v*) of the stock solution). Figure S5: Calibration curves (concentration range from 0.3 to 9.1 ng/mL) obtained with mixed standard solutions of T-2, HT-2, T2-glucoside and HT2-glucoside (expressed as sum, ratio 1:1:0.5:0.5) (black square) and spiked diluted extracts of wheat, obtained using Protocol A (a) and Protocol B (b), by analyzing 5 mg (multiplication sign), 10 mg (white triangle) and 20 mg (black circle) of matrix equivalent.
