# Supplementary material for: Fluorescence Polarization Immunoassay for the Determination of T-2 and HT-2 Toxins and Their Glucosides in Wheat
Source: Toxins (Basel). 2019 Jul 1;11(7):380. doi: 10.3390/toxins11070380 (PMC6669535; doi:10.3390/toxins11070380)
Supplement: Supplementary file 1 [file toxins-11-00380-s001.pdf]

Supplementary Materials: Fluorescence Polarization Immunoassay for the Determination of T-2 and HT-2 Toxins and Their Glucosides in Wheat

Vincenzo Lippolis *, Anna C. R. Porricelli, Erminia Mancini, Biancamaria Ciasca, Veronica M.T. Lattanzio, Annalisa De Girolamo, Chris M. Maragos, Susan McCormick, Peiwu Li, Antonio F. Logrieco and Michelangelo Pascale

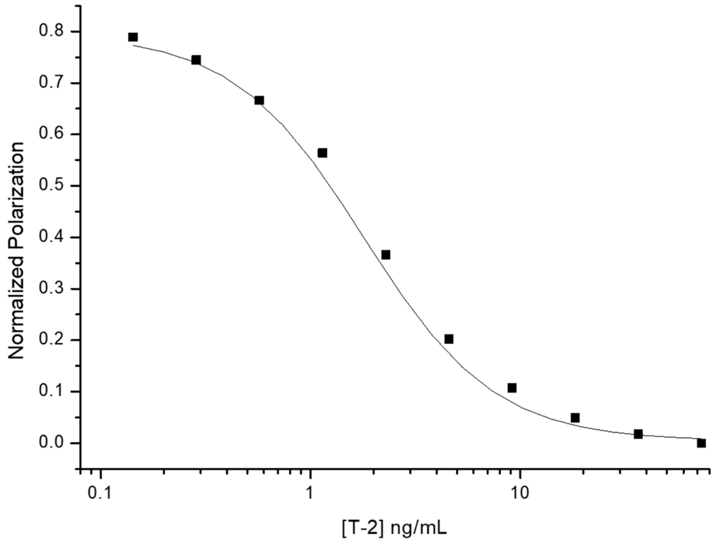

Figure S1. Normalized calibration curve of the selected FPIA obtained with mixed standard solutions of T-2 toxin (T-2) in PBS-A solution ([Anti-HT2] = 8 µg/mL; [HT2-FL_{1a}] obtained after dilution 1:3000 (*v*/*v*) of the stock solution). Values of *x*-axes are relevant to toxin concentrations in the final test solution.

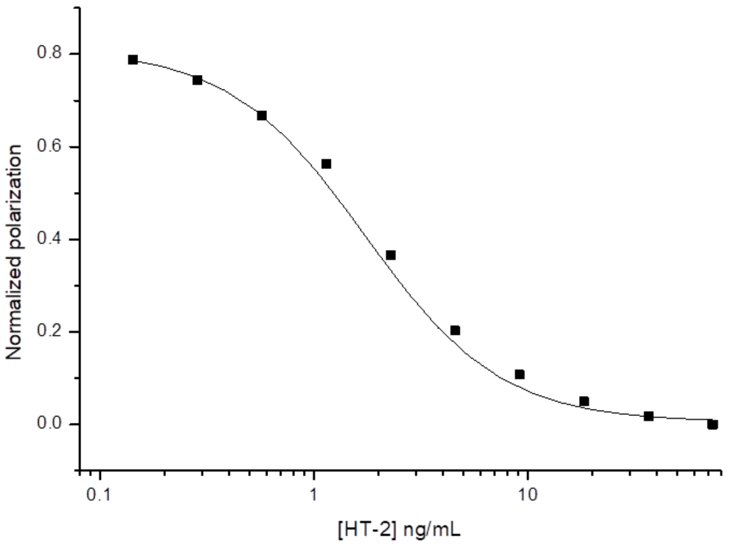

Figure S2. Normalized calibration curve of the selected FPIA obtained with mixed standard solutions of HT-2 toxin (HT-2) in PBS-A solution ([Anti-HT2] = 8 µg/mL; [HT2-FL_{1a}] obtained after dilution 1:3000 (*v*/*v*) of the stock solution). Values of *x*-axes are relevant to toxin concentrations in the final test solution.

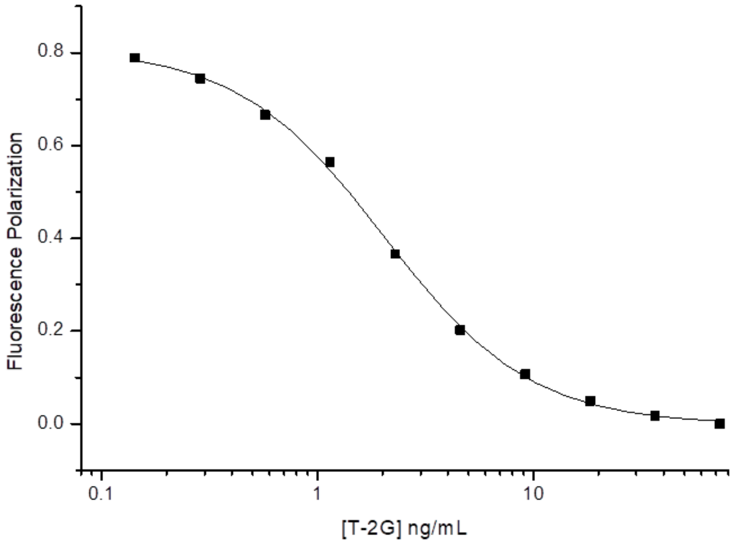

Figure S3. Normalized calibration curve of the selected FPIA obtained with mixed standard solutions of T-2 glucoside (T-2G) in PBS-A solution ([Anti-HT2] = 8 µg/mL; [HT2-FL_{1a}] obtained after dilution 1:3000 (*v*/*v*) of the stock solution). Values of *x*-axes are relevant to toxin concentrations in the final test solution.

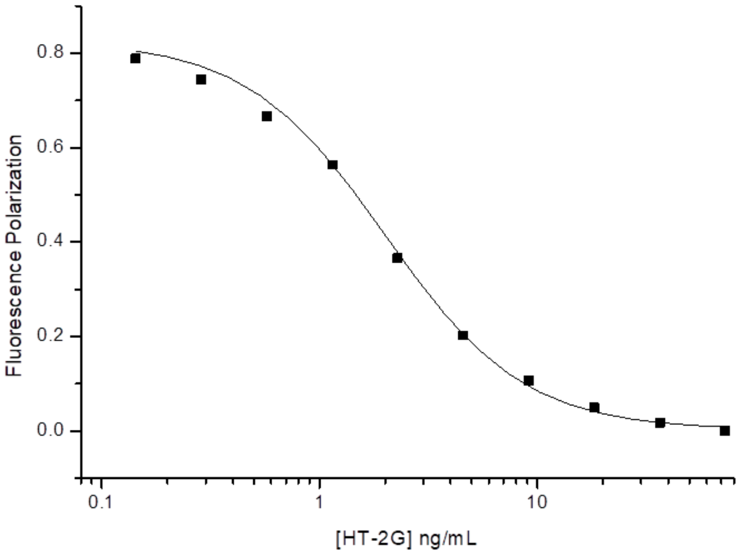

Figure S4. Normalized calibration curve of the selected FPIA obtained with mixed standard solutions of HT-2 glucoside (HT-2G) toxin in PBS-A solution ([Anti-HT2] = 8 µg/mL; [HT2-FL_{1a}] obtained after dilution 1:3000 (*v*/*v*) of the stock solution). Values of *x*-axes are relevant to toxin concentrations in the final test solution.

(a)

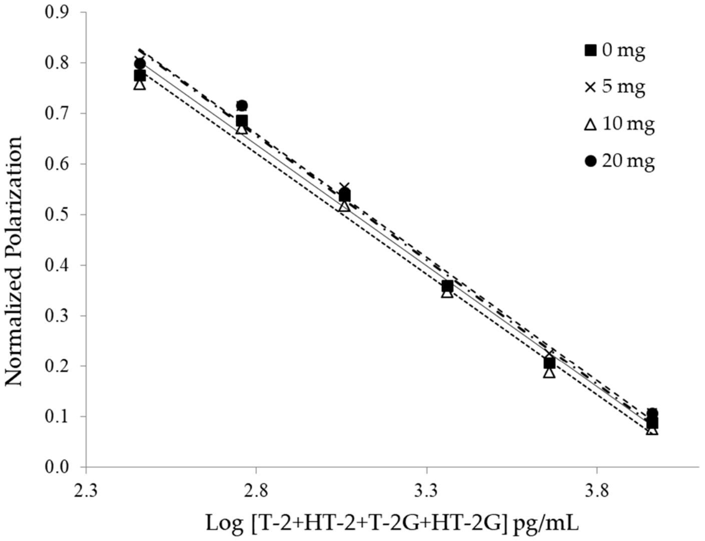

(b)

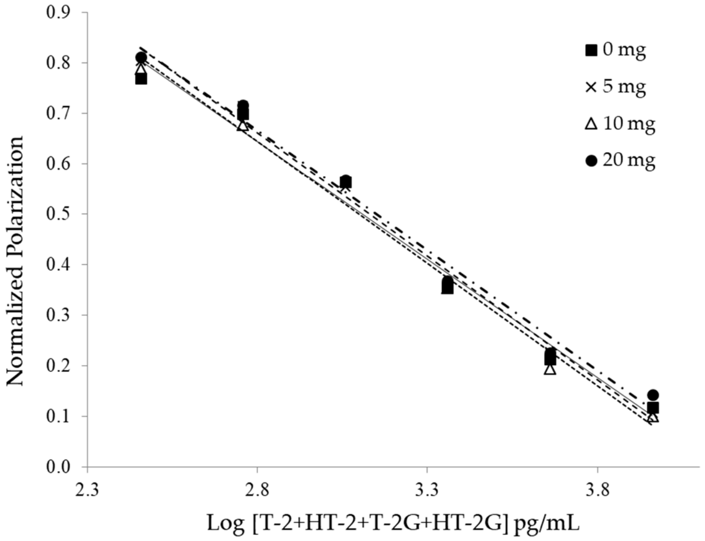

Figure S5. Calibration curves (concentration range from 0.3 to 9.1 ng/mL) obtained with mixed standard solutions of T-2, HT-2, T2-glucoside and HT2-glucoside (expressed as sum, ratio 1:1:0.5:0.5) (black square) and spiked diluted extracts of wheat, obtained using Protocol A (**a**) and Protocol B (**b**), by analyzing 5 mg (multiplication sign), 10 mg (white triangle) and 20 mg (black circle) of matrix equivalent.
